# The arrow of time in Parkinson’s disease

**DOI:** 10.1016/j.nicl.2025.103834

**Published:** 2025-06-24

**Authors:** Fatemeh Sadeghi, Elvira del Agua Banyeres, Alessandra Pizzuti, Abdullah Okar, Kai Grimm, Christian Gerloff, Morten L. Kringelbach, Rainer Goebel, Simone Zittel, Gustavo Deco

**Affiliations:** a Department of Neurology, University Medical Center Hamburg-Eppendorf, 20246 Hamburg, Germany; b Center for Brain and Cognition, Computational Neuroscience Group, Universitat Pompeu Fabra, 08005 Barcelona, Spain; c Department of Cognitive Neuroscience, Faculty of Psychology and Neuroscience, Maastricht University, 6211 LK Maastricht, the Netherlands; d Brain Innovation B.V., 6229 EV Maastricht, the Netherlands; e Centre for Eudaimonia and Human Flourishing, Linacre College, University of Oxford, Oxford OX1 3JA, UK; f Life and Health Sciences Research Institute (ICVS), School of Medicine, University of Minho, 4710-057 Braga, Portugal; g Center for Music in the Brain, Department of Clinical Medicine, Aarhus University, 8000 Aarhus, Denmark; h Department of Psychiatry, University of Oxford, Oxford OX3 7JX, UK; i Department of Information and Communication Technologies, Universitat Pompeu Fabra, 08018 Barcelona, Spain; j Institució Catalana de la Recerca i Estudis Avancats (ICREA), 08010 Barcelona, Spain

**Keywords:** Arrow of time, Brain dynamics, Equilibrium, Hierarchical organization, Non-reversibility, Parkinson’s disease

## Abstract

**Background:**

Parkinson’s disease (PD) is a system-level disorder that implicates brain network dynamics across multiple scales. Detecting the ‘arrow of time’, or temporal reversibility of the brain’s information processing flow enables quantification of equilibrium in the brain and inferences on the hierarchical organization. Therefore we aimed to explore disturbances in resting-state equilibrium levels as well as changes in the hierarchical organization due to PD.

**Methods:**

Structural and functional MRI of 29 PD patients and 19 healthy controls were acquired and analyzed. Empirical non-reversibility was computed as the distance between time-shifted forward- and artificially-reversed time series. Levels of equilibrium were subsequently assessed globally and within two cortico-subcortical motor networks implicated in PD. Moreover, whole-brain generative computational models consisting of 1051 Hopf oscillators were constructed to evaluate effective connectivities and alterations of the functional hierarchical organization.

**Results:**

We found that PD is characterized by disrupted equilibrium regimes, marked by distinct effective connectivity patterns, particularly within the motor networks. Additionally, we observed a flatter hierarchical organization in PD, with the cerebellum and thalamus exerting increased influence.

**Conclusion:**

The arrow of time methodology effectively identifies distinct and informative characteristics of PD. Our analyses suggest that PD shifts the brain towards less efficient, non-equilibrium dynamics that impair intrinsic flexibility and disrupt motor coordination. Thus, these findings not only provide insight into widespread system alterations in PD that could serve as potential biomarkers, but also lay the groundwork for next-generation stimulation techniques aimed at restoring balance in the Parkinsonian brain.

## Highlights

• The arrow of time reveals temporal reversibility and brain equilibrium using fMRI.
• Parkinson’s disease disrupts equilibrium, leading to less efficient brain dynamics.
• The imbalance is especially visible in cortico-subcortical motor networks.
• Functional hierarchy is flatter in PD, altering cerebellum and thalamus roles.
• These altered dynamics may serve as PD biomarkers and guide brain stimulations.

## Graphical abstract

**Figure:**
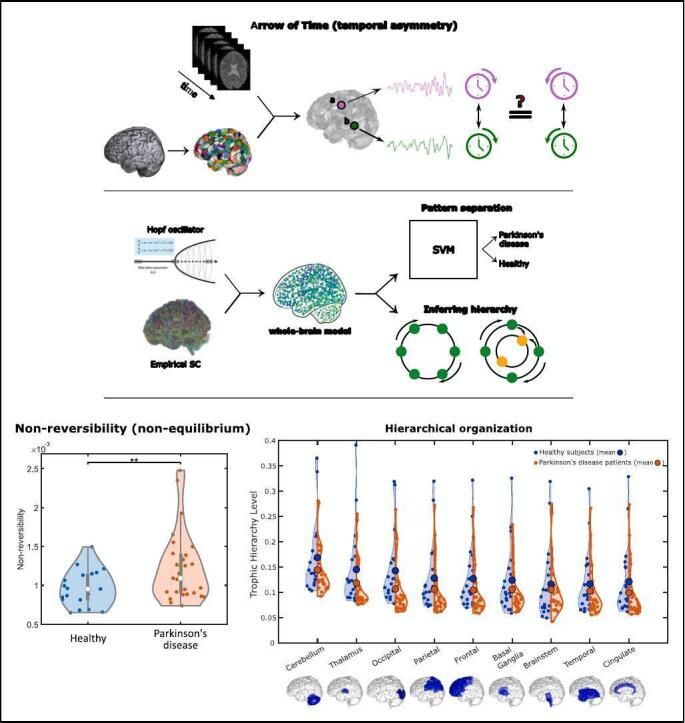

## 1 Introduction

*“The second law of thermodynamics opens up a new province of knowledge, namely, the study of organisation; and it is in connection with organisation that a direction of time-flow and a distinction between doing and undoing appears for the first time“.*

*Arthur Eddington (*Murphy and Eddington 1928*)*.

The human brain relies on time-sensitive network-level information processing, supported by the formation of spatiotemporal patterns (Carr, 1993, Buzsáki, 2009, Deco and Jirsa, 2012). The ‘arrow of time’ – a concept introduced by Sir Arthur Eddington and rooted in thermodynamics – offers a powerful framework for studying these brain dynamics through the lens of temporal reversibility, revealing changes in whole-brain information flow (Murphy and Eddington 1928).

In a system at thermodynamic equilibrium, dynamics obey detailed balance: the probability of transitioning from one state to another is equal to that of the reverse, producing temporal reversibility. In contrast, non-equilibrium systems break this symmetry, resulting in a preferred direction of flow and the macroscopic production of entropy (Geli et al. 2025). The degree of deviation from equilibrium can be quantified by comparing the observed process to its time reversal. In the brain, this is reflected in temporal asymmetry—also known as non-reversibility—which serves as an indirect and scalable proxy for non-equilibrium and entropy production (Lynn et al., 2021, Lynn et al., 2022).

The brain, as a complex self-organized system, operates in a constant state of non-equilibrium, marked by continuous energy consumption and inherently non-reversible molecular and cellular processes (Tomé and De Oliveira, 2012, Lynn et al., 2021, Sanz Perl et al., 2021). Furthermore, to support efficient large-scale communication, brain regions operate within a specific functional hierarchical organization, where directional influences define the ranking of interconnected regions (Buzsáki, 2009, Shettigar et al., 2022). These ranks can be quantified based on asymmetries between the in- and out-flow of information among pairs of regions (Mackay et al., 2020, Kringelbach et al., 2023).

While traditional brain network measures such as functional connectivity (FC) quantify statistical dependencies between regions, they do not capture the temporal direction or asymmetry of interactions. In contrast, non-reversibility reveals the arrow of time by comparing forward and time-reversed dynamics, thereby exposing violations of detailed balance and directional information flow. This approach builds directly on the same BOLD signals as FC, but extends beyond static correlations by capturing temporal asymmetries in inter-regional interactions, offering sensitivity to dynamic imbalances and directional disruptions that may go undetected by conventional measures, particularly in pathological conditions.

Seif et al. (2021) introduced a machine-learning approach to quantify time irreversibility in complex systems. Building on this foundation, Deco et al. (2022) developed a framework to detect temporal non-reversibility in brain activity using resting-state functional magnetic resonance imaging (fMRI) signals. In this method, time-lagged correlations between pairs of brain regions are computed in both the original and time-reversed BOLD signals, with asymmetries between them serving as proxies for directional information flow (Shettigar et al. 2022). In a healthy non-equilibrium regime, the flow of information exhibits characteristic asymmetries. Deviations from this regime, marked by altered asymmetries, indicate a disruption in the system’s dynamic balance and may reflect disease-related changes in large-scale brain coordination (Deco et al., 2021, Cruzat et al., 2023). Numerous studies have explored applications of the arrow of time and non-reversibility to study and differentiate brain states, including neurological disorders, and investigate their underlying mechanisms (Zanin et al., 2020, Sanz Perl et al., 2021, Bolton et al., 2023, Cruzat et al., 2023, de la Fuente et al., 2023, G-guzmán et al., 2023, Kringelbach et al., 2023).

Recent advances have further extended this framework through whole-brain computational modeling. Notably, generative models such as the Hopf oscillator model (Deco and Kringelbach, 2014, Kringelbach et al., 2023) enable the estimation of effective connectivity patterns by jointly fitting structural connectivity (from diffusion MRI), functional connectivity (from BOLD signals), and lagged covariances encoding non-reversibility. This approach offers a principled way to infer the directed coupling structure of the brain while preserving key empirical constraints. Such models have shown growing promise in uncovering latent features of brain dynamics, improving our understanding of systems-level disruptions in disease, and advancing biomarker discovery (Schiff, 2010, Deco and Kringelbach, 2014, Little and Bestmann, 2015, Breakspear, 2017, Humphries et al., 2018, Makarious et al., 2022).

Parkinson’s disease (PD), the second-most common neurodegenerative disorder, is associated with network-level impaired structure and functional connectivity (Caligiore et al., 2016, Poewe et al., 2017). While PD originates from the degeneration of dopamine-producing neurons in the substantia nigra, its impact gradually propagates across broader brain systems, disrupting the balance and integrity of motor circuits, particularly the basal ganglia–thalamo–cortical (BTC) and cerebello–thalamo–cortical (CTC) loops. These widespread alterations destabilize the fine-tuned coordination of activity across regions, contributing to hallmark motor symptoms such as bradykinesia, tremor, postural instability, and gait disturbances (Poewe et al., 2017, Helmich, 2018, Dirkx and Bologna, 2022).

Although structural and functional impairments in PD have been well-documented (van Eimeren et al., 2009, Delaveau et al., 2010, Tahmasian et al., 2015, Kim et al., 2017, Filippi et al., 2019, Wolters et al., 2019, Filippi et al., 2021), the broader dynamical consequences of the disease remain less well understood. In particular, the extent to which PD shifts the brain’s intrinsic activity away from equilibrium — disrupting the directional coordination of information flow — is not yet established. Identifying system-level markers of such non-equilibrium could provide a new dimension for understanding disease mechanisms and progression (Miller and O’Callaghan, 2015, Caligiore et al., 2016, Obeso et al., 2017, Tolosa et al., 2021).

Building on this foundation, we hypothesize that PD disrupts the intrinsic balance of brain dynamics by altering the directionality and coordination of information flow across regions. These disruptions are expected to manifest as increased temporal non-reversibility, reflecting deviations from the baseline non-equilibrium dynamics that characterize healthy resting-state activity. Given the progressive disintegration of connectivity in PD, particularly within the BTC and CTC circuits, we anticipate that such deviations will be detectable across multiple spatial scales — from local regions to networks and ultimately the whole brain.

To test this hypothesis, we first quantified non-reversibility empirically from resting-state fMRI in PD patients and healthy controls. We then extended the analysis using subject-specific whole-brain computational models, which jointly incorporated individual structural connectivity, functional connectivity, and lagged covariances encoding non-reversibility. These models allowed us to evaluate how well temporal asymmetry captures PD-related changes in effective connectivity. Finally, we explored how these disruptions alter the brain’s functional hierarchical organization, revealing how the relative influence among regions may be changed in the PD state.

## 2 Materials and methods

### 2.1 Participants

Thirty individuals diagnosed with PD (mean age 60 years, SD = 10.80) were recruited from the outpatient clinic of the Department of Neurology of the University Medical Center Hamburg-Eppendorf, plus twenty healthy age- and sex-matched participants (mean age 64 years, SD = 9.02). Sex was defined as the biological attribute assigned at birth (male/female) based on external anatomy. No participants identified as intersex or reported differences of sex development (DSD). Gender identity was not assessed in this study. The inclusion criteria were as follows: (1) age between 40 and 85 years; (2) diagnosis of PD according to Parkinson’s disease UK Brain Bank criteria (for patients only) (Litvan et al. 2003); (3) no history of head trauma, coexistence of other neurological disorders, psychiatric illness, or substance abuse; (4) compliance with MRI safety standards. Patients underwent clinical assessments and MRI scans in the OFF state, with PD medication discontinued for a minimum of 12 h before the experiment. The privacy rights of all participants were respected, and written informed consent was obtained from each individual.. All experiments in this study adhered to the Declaration of Helsinki and relevant laws and institutional guidelines and have been approved by the Hamburg local ethics committee with ID number 2020–10281-BO-ff.

### 2.2 Clinical Assessment

The patients underwent a clinical neurological examination by a board-certified neurologist with specialty training in movement disorders (SZ) who was blinded to the MRI data. Motor symptom severity was assessed using the Movement Disorder-Unified Parkinson’s Disease Rating Scale (MDS-UPDRS) parts II and III (Goetz et al. 2008), and Hoehn and Yahr stages (Hoehn and Yahr 1967).

### 2.3 MRI acquisition

All participants underwent the same MRI scanning sequences. T1-weighted MRI images were acquired via a magnetization prepared-rapid gradient echo (MPRAGE) sequence on a 3 T scanner (Siemens MAGNETOM Prisma, Erlangen, Germany) with a standard 64-channel head coil. Sequence parameters: 256 coronal slices, field of view (FOV) = 230 mm, repetition time (TR) = 2500 ms, echo time (TE) = 2.15 ms, flip angle = 8°, voxel size = 0.8 x 0.8 x 0.8 mm, matrix dimension = 232 x 288 x 256, scanning time = 5′:49′’, and bandwidth = 240 Hz/pixel. For resting-state functional images, gradient echo planar imaging (EPI) sensitive to BOLD contrast was used. Resulting in 200 coronal slices, FOV = 216 mm, TR = 2220 ms, TE = 30 ms, flip angle = 80°, voxel size = 3 x 3 x 3 mm, matrix dimension = 504 x 504 x 200, distance factor 20 %, scanning time = 7′:32′’, and bandwidth = 2170 Hz/pixel. Diffusion-weighted imaging (DWI) acquisition was performed using 96 optimal nonlinear diffusion gradient directions at b = 0 and b = 2000 s/mm^2^. Parameters: FOV = 218 mm, TR = 6800 ms, TE = 76 ms, voxel size = 1.8 x 1.8 x 1.8 mm, matrix size = 122 x 122 x 480, and bandwidth of 1640 Hz/pixel.

### 2.4 MRI processing

Anatomical T1-w images were visually controlled for quality, focusing on detecting and correcting artifacts due to tremor and head motion in PD patients. The artifact-free images were further processed using the Connectome Mapper 3 (v3.1.0) (Tourbier et al. 2022) and Freesurfer (v7.1.1) for normalization, segmentation, and cortical surface reconstruction (Desikan et al. 2006). The processed images were parcellated into 1058 regions of interest (ROI) using the Lausanne2018 atlas, encompassing 998 cortical and 60 subcortical regions (Cammoun et al., 2012, Tourbier et al., 2022). To include the cerebellum in the parcellation, it was separately isolated and segmented into thirteen lobules using the automatic Cerebellums Segmentation (CERES) pipeline (Romero et al., 2017, Carass et al., 2018). The segmented volume was then integrated into the Lausanne2018 parcellation, creating a whole-brain parcellation with 1084 ROIs, referred to as LC.

To facilitate regional analysis, the 1084 parcellated regions were grouped into: cortical lobes, thalamus, basal ganglia, brainstem, and cerebellum, based on the Desikan-Killiany mapping (Alexander et al. 2019). For the network analyses, ROIs belonging to BTC and CTC networks were extracted following delineations by Caligiore et al. (2016) and Lewis et al. (2013), respectively. The BTC network included 167 ROIs in the basal ganglia (striatum, globus pallidus internus and externus, and subthalamic nucleus), thalamus (anterior ventral lateral nucleus), and motor cortex (primary and supplementary motor area). The CTC network included 255 ROIs in the cerebellum, thalamus (posterior ventral lateral nucleus), and motor cortex (premotor and somatosensory areas). Furthermore, a ‘no-network’ parcellation was created, containing the remaining 751 ROIs outside the BTC and CTC.

Certain ROIs, such as the primary motor cortex, were defined based on a functional atlas, while the LC parcellation relied on anatomical annotations. To align these, subject-specific functional parcellations were generated according to Rolls et al. (2020) and matched to the LC parcellation through a custom optimum probability mapping algorithm in MATLAB (R2022b, The MathWorks, Inc.). Details are provided in the Supplementary Material: Bridging parcellations.

The processing of DWI images involved denoising with Mrtrix3 (Tournier et al. 2019); correction of magnetic resonance bias field effects using FSL FAST, followed by correction of motion artifacts and eddy currents using FSL MCFLIRT and Eddy respectively (https://www.fMRIb.ox.ac.uk/fsl, FMRIB, Oxford). The preprocessed data was resampled to 1 x 1 x 1 mm voxels and co-registered to T1-w images using the ANTS toolbox (Avants et al. 2009). Reconstruction and tractography were performed based on probabilistic models of Mrtrix3, leading to the computation of subject-specific structural connectivity (SC) matrices.

fMRI preprocessing involved discarding the first five volumes, slice timing, and linear head motion corrections using FSL, and linear trend removal using scipy library. Afterward, motion-, CSF-, and WM-induced nuisance signals were regressed out using the general linear model method. Resting-state fMRI images were then co-registered to T1-w using FSL, and ROI-averaged BOLD time series were extracted for each participant. Diffusion and fMRI pipelines were conducted based on individual LC parcellations in native space.

### 2.5 Empirical framework of non-reversibility/non-equilibrium

To quantify non-reversibility from empirical MRI data, we adopted the INSIDEOUT framework, which estimates the degree of temporal asymmetry in brain dynamics as an indirect measure of non-equilibrium (Deco et al., 2022, Kringelbach et al., 2023, Geli et al., 2025). Specifically, we assessed whether the directionality of pairwise interactions between brain regions—approximated through time-lagged correlations—remained symmetric when time was reversed.

**Fig. 1:**
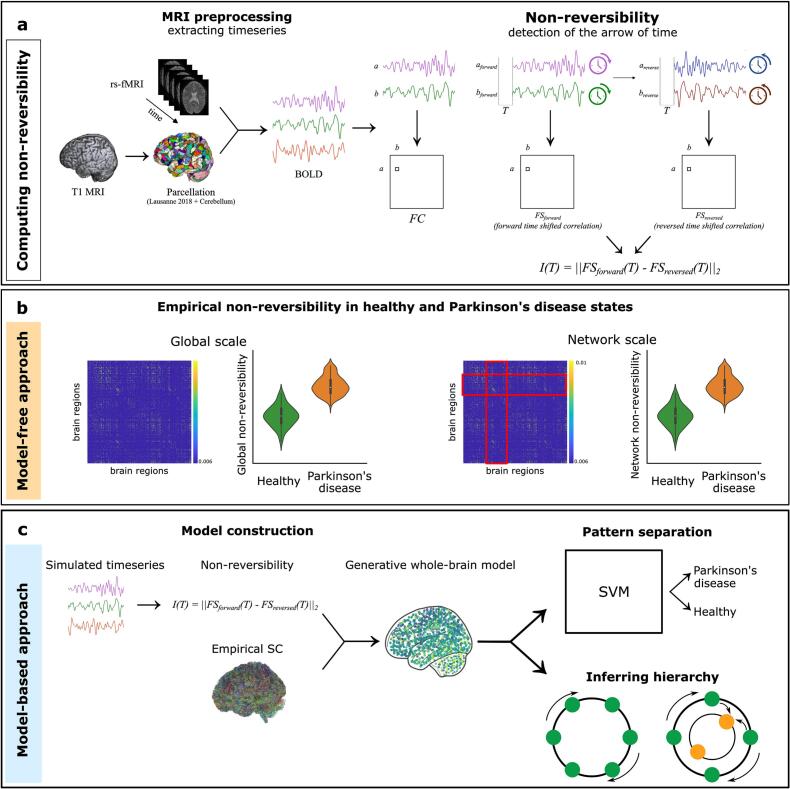
**Methodological workflow of capturing the arrow of time and its application in studying Parkinson’s disease (PD). (a)** The step-wise technique of computing non-reversibility (non-equilibrium). The anatomical T1-w images are processed and parcellated into a thousand regions of interest (ROI) in cortical, subcortical, and cerebellar regions. The Resting-state fMRI data are processed and BOLD signals are extracted from each ROI. Levels of non-reversibility are computed similarly to the FC matrix (pairwise correlations between BOLD signals), however by computing pair-wise correlations between time-shifted forward and artificially reversed time series, non-reversibility or asymmetry of the flow of information processing can be revealed. The resulting non-reversibility level indicates deviations from equilibrium and entropy production. **(b)** The model-free INSIDEOUT approach allows empirical non-reversibility measurement on multiple spatial scales. **(c)** Via the model-based approach, whole-brain computational models of generative effective connectivity (GEC) are constructed and informed by empirical non-reversibility levels, thus providing insight into hierarchical organization alterations between healthy and PD states.

As outlined in Fig. 1a-b, for each pair of brain regions $x_{i}$ and $x_{j}$, BOLD time series $x_{i}\left(t\right)$ and $x_{j}\left(t\right)$ were extracted and artificially reversed to obtain ${x_{i}}^{\left(r\right)}\left(t\right)$ and ${x_{j}}^{\left(r\right)}\left(t\right)$. For both the original and reversed data, we computed time-lagged Pearson correlations between region pairs, which we interpreted as proxies for directed information flow, as given by:

(1) $${\mathrm{FS}}_{forward,ij}\left(\Delta t\right)=-\frac{1}{2}log\left(1-\;{\langle x_{i}\left(t\right)\;,\;x_{j}\left(t+\Delta t\;\right)\rangle }^{2}\right)$$

(2) $${\mathrm{FS}}_{reversed,ij}\left(\Delta t\right)=-\frac{1}{2}log\left(1-\;{\langle x_{i}^{\left(r\right)}\left(t\right)\;,\;x_{j}^{\left(r\right)}\left(t+\Delta t\;\right)\rangle }^{2}\right)$$

The notation 〈〉 indicates the time-average Pearson correlation between two time series. Expanding equations Eqn (1), (2) across the whole brain for all region pairs, we can capture the directional dependencies ${\mathrm{FS}}_{forward}$ and ${\mathrm{FS}}_{reversed}$. It is important to note that this approximation is to ensure the output is positive and interpretable as a measure of functional dependency and does not imply any Gaussian assumption about the underlying brain signals or their distributions. All correlation values are computed directly from the empirical BOLD data without assuming any particular distributional form.

For a given time shift Δt=T, the asymmetry between these matrices reflects violations of detailed balance and thus the system’s deviation from equilibrium. We quantified this asymmetry using the squared Frobenius norm:

(3) $$I={\|{\mathrm{FS}}_{\mathrm{forward}}\left(T\right)-\;{\mathrm{FS}}_{\mathrm{reversed}}\left(T\right)\|}_{2}$$

A time shift of $T=1TR\;\left(2.2seconds\right)$ was selected, ensuring sufficient temporal separation for directional comparisons while remaining within the autocorrelation decay range of the BOLD signal. From the pre-processed fMRI data, BOLD signals from 1084 ROIs were extracted, temporally detrended, and band-pass filtered between 0.008 and 0.08 Hz. Twenty-two hippocampal regions and eleven gyral subregions were excluded uniformly across subjects due to insufficient time series quality and non-reversibility matrices were computed for the remaining 1051 nodes.

To compare global non-reversibility (non-equilibrium) between PD and healthy controls, we averaged all elements of the I matrix to obtain a single global value per subject. For network-level analysis, non-reversibility values within the BTC and CTC networks were averaged and compared between cohorts. At the node level, the I matrix was flattened into a single-dimensional long array for each subject, and a linear mixed model (LMM) were used to compare non-equilibrium levels between groups, accounting for individual variability while controlling for age and sex. Finally, group differences in the spatial distribution of node-level non-reversibility were assessed using two non-parametric methods: the squared Maximum Mean Discrepancy (MMD^2^) (Gretton et al. 2012) and the minimum energy test (Aslan and Zech, 2005 with 1000 permutations. These approaches are robust to differences in shape, scale, and higher-order moments, and are well suited for comparing multivariate distributions without assuming normality.

To explore the clinical relevance of altered brain dynamics, we additionally tested the association between non-reversibility values and clinical manifestation of PD. Specifically, we examined correlations between global and network-level non-reversibility measures and motor symptom severity (MDS-UPDRS part III) as well as disease stage (Hoehn and Yahr score) using Spearman’s rank correlation.

### 2.6 Model-based framework of non-reversibility

To complement our empirical findings and investigate the dynamical mechanisms underlying altered brain states in PD, we extended our analysis by constructing subject-specific whole-brain computational models. These models enabled us to simulate the directional information flow across brain regions and to examine whether the empirically observed non-reversibility can be explained by underlying disruptions in effective connectivity and hierarchical organization. A summary of the model-based methodology is presented in Fig. 1c.

This modeling step served two primary goals: (i) allowing us to reproduce empirical functional dynamics using an interpretable generative framework, and (ii) providing access to latent directed interactions such as effective connectivity which are not directly observable from BOLD correlations alone.

#### 2.6.1 The Hopf model

We used individual whole-brain models based on coupled Hopf oscillators, which are well-suited for modeling large-scale brain dynamics due to their ability to capture multistability, oscillatory regimes, and noise-driven transitions—all features relevant to resting-state activity and known to be altered in PD (Cabral et al., 2011, Deco et al., 2017, Saenger et al., 2017). Each brain region (node) is represented by a linearized Hopf oscillator, which can represent a state between noise-dominated and oscillatory behavior depending on a bifurcation parameter. Nodes are anatomically coupled via subject-specific structural connectivity, and their interactions give rise to emergent whole-brain activity patterns.

Following the framework proposed by Kringelbach et al. (2023), we constructed generative effective connectivity (GEC) models, representing the brain as a directed network of N coupled oscillators, with each node’s dynamics simulated by a linearized version of the Hopf oscillator (Ponce-Alvarez and Deco 2024). The local dynamics of node j are governed by the following differential equation.

(4) $$\frac{dz_{j}}{\mathrm{dt}}=z_{j}\left(a_{j}+i\omega_{j}\;-\left|z_{j}^{2}\right|\right)+\sum_{k=1}^{N}C_{\mathrm{jk}}\left(z_{k}-z_{j}\right)+\eta_{j}$$

Where $z_{j}=\rho_{j}e^{i\theta_{j}}=x_{j}+iy_{j}$ is the complex state variable of node j, $\omega_{j}$ represents the intrinsic frequency of the oscillator derived from the average peak frequency of the empirical BOLD time series, and $C_{\mathrm{jk}}$ denotes the coupling strength from node k to j, and $\eta_{j}$ is the additive Gaussian noise (standard deviation = 0.02). The parameter $a_{j}$ controls the dynamical regime: for $a_{j}<0$ there is a stable fixed point at $z_{j}=0$; at $a_{j}=0$ the system has a bifurcation, and for $a_{j}>0$ the dynamics exhibit limit cycle oscillations with a frequency of $\frac{\omega_{j}}{2\pi }$ Hz. In our study, a fixed value of $a_{j}=-0.02$ was used for all nodes, consistent with resting-state activity and previous findings (Deco et al. 2017).

Due to the high dimensionality of the parcellation (1051 ROI), training individual GEC models for each subject proved computationally impractical. To address this, a linear approximation technique was employed under assumptions of small nonlinearities and weak noise Deco et al., 2023b. This method directly estimates system statistics without requiring computationally intensive BOLD data simulations by neglecting higher-order dynamical terms, which have been shown to exert minimal influence on non-equilibrium behavior in large-scale systems near equilibrium (Geli et al. 2025). More details are provided in the Supplementary Material: The Hopf model.

The model fitting relied on two complementary measures: the functional connectivity (FC) matrix, capturing pairwise static correlations between brain regions, and the time-lagged shifted covariance matrix ${\mathrm{CS}}_{v}\left(T\right)$, which is a linear representation of $FS\left(T\right)$, to capture dynamic non-linear dependencies between brain regions. Model fitting was guided by the following update rule for a specified time shift Δt=T:

(5) $$C_{i,j}=C_{i,j}+∊\left({\mathrm{FC}}_{i,j}^{\mathrm{empirical}}-\;{\mathrm{FC}}_{i,j}^{\mathrm{model}}\right)+∊\prime \left({{\mathrm{CS}}_{v}\left(T\right)}_{i,j}^{\mathrm{empirical}}-\;{{\mathrm{CS}}_{v}\left(T\right)}_{i,j}^{\mathrm{model}}\right)$$

Two learning rates ∊=0.0004 and ∊′=0.0001 were considered. To enhance the training process, a mean C was first trained from each group’s mean empirical FC and ${\mathrm{CS}}_{v}\left(T\right)$, followed by initializing individual C values with the relative group means and further optimizing until convergence, to finally obtain a directed GEC matrix for each subject. To ensure that the optimization process accurately represented the anatomical features of each subject's brain, only nodes with non-zero connecting fiber density values—identified via the normalized SC matrix—were included. To examine differences in directed GEC graphs between healthy individuals and PD patients, we calculated node strengths and compared it between cohorts (details in Supplementary Material: Comparing GEC Node Strengths). An LMM was further used to compare node strengths between cohorts, adjusting for age, sex, and subject variability.

#### 2.6.2 Pattern separation

To assess the informative and distinctive qualities of the directed GEC matrices in PD, we conducted a pattern separation analysis between the two cohorts. We selected a support vector machine (SVM) due to its effectiveness in handling large data, robustness against overfitting, and to avoid issues related to multiple comparisons. We trained a linear kernel SVM to differentiate the GEC patterns between the healthy and PD cohorts. Cross-validation, with an 85 % to 15 % training-to-test ratio across 1000-fold iterations, ensured robustness. To validate SVM outputs, we extended pattern separation to SC and FC. Network-based analyses focused on the BTC and CTC loops. As a control measure for the network analysis, models with regions outside of these networks were subjected to the same pattern separation algorithm.

#### 2.6.3 Inferring the brain’s hierarchical organization

The obtained GEC models are whole-brain graphs depicting directed, weighted dynamic connections among anatomically connected regions. These directed connections indicate effective connectivity and reveal information flow between nodes—nodes receiving more input from others tend to be more influential within the network. By quantifying the asymmetry between incoming and outgoing flows across the entire brain using the method described by Mackay et al. (2020), otherwise known as trophic hierarchical organization analysis, we ranked nodes based on their functionality as well as influential relationships among major brain regions,

From the GEC model, with N nodes and E directed edges, an edge from node m to node n is denoted m→n, with weight $\omega_{\mathrm{mn}}>0$. These weights form a matrix W, where $\omega_{\mathrm{mn}}=0$ indicates no edge from m to n. When edge weights are set to 1, this matrix becomes the adjacency matrix A. Multiple edges between m and n are summed, while self-edges (m→m) are allowed. For each node n, in-weight and out-weight (or in-strength and out-strength) are defined as follows:

(6) $$\omega_{n}^{\mathrm{in}}=\;\sum_{m\in N}\omega_{\mathrm{mn}}\;and\omega_{n}^{\mathrm{out}}=\;\sum_{m\in N}\omega_{\mathrm{nm}}$$

The total weight of the node n is defined by $u_{n}$ as per Eqn 8.

(7) $$u_{n}=\;\sum_{m\in N}\omega_{\mathrm{mn}}+\;\sum_{m\in N}\omega_{\mathrm{nm}}$$

And the imbalance for node n is given by $v_{n}$ which represents the difference between the in- and out-flow of the node:

(8) $$v_{n}=\;\omega_{n}^{\mathrm{in}}-\;\omega_{n}^{\mathrm{out}}$$

The weighted graph-Laplacian operator Λ on vectors h is defined by:

(9) $${\left(\Lambda h\right)}_{m}=u_{m}h_{m}-\sum_{n\in N}\left(\omega_{\mathrm{mn}}+\omega_{\mathrm{nm}}\right)h_{n}$$

The matrix representation, with W denoted as the matrix form of ω, is provided in Eqn 11.

(10) $$\Lambda =diag\left(u\right)-W-\;W^{T}$$

The hierarchical rank is defined as the solution of h of the linear system of equations as given below.

(11) Λh=v

Upon determining the hierarchy level h, we computed the global incoherence, or the directionality of the network as per Eqn 13

(12) $$F_{0}=\frac{\sum_{\mathrm{mn}}\omega_{\mathrm{mn}}\;{\left(h_{n}-h_{m}-1\right)}^{2}}{\sum_{\mathrm{mn}}\omega_{\mathrm{mn}}\;}$$

Coherence is quantified by $1-F_{0}$, where$F_{0}$ = 0 indicates maximal coherence and$F_{0}$ = 1 denotes complete incoherence. Using directed GEC models, we computed whole-brain coherence and hierarchical levels for the 1051-ROI parcellation in PD and healthy states. More details are provided in Supplementary Material: Global Coherence.

Hierarchical organization flatness was compared between states by analyzing the dispersion of rank distributions via standard deviation. Node-level hierarchical ranks were compared using LMMs to account for individual variability. At the network scale, hierarchy levels were averaged within broader brain regions, including cortical lobes, thalamus, basal ganglia, brainstem, and cerebellum, by averaging ROI hierarchy levels within each region. Since the relative differences between hierarchy levels indicate the influence of one region over another, we performed pairwise comparisons of regional influences across all regions, separately for PD and healthy states according to Mackay et al. (2020). Furthermore, PageRank centrality was computed for each ROI (Lawrence et al. 1990), with ROIs ranked both by centrality and hierarchical levels. Details are provided in Supplementary Material: Centrality measures in the Generative effective connectivity graph.

### 2.7 Statistical analysis

Demographic disparities were assessed between PD and healthy cohorts using two-tailed, t-tests for continuous variables such as age, and the Kruskal–Wallis tests for categorical variables such as sex. Global and network non-reversibility, global coherence, and hierarchical levels were compared between PD and healthy groups using Wilcoxon rank-sum tests to account for non-normality and variance heterogeneity. To evaluate variance in hierarchical organization, the Mann-Whitney *U* test was employed, as it is particularly suited for comparing distributions between two groups without assuming normality or equal variances. Node-level analyses of non-reversibility, GEC, and hierarchy levels employed LMMs with cohort as the primary variable, controlling for age and sex, with subject ID as a random effect. An alpha of 0.05 was applied, and results were FDR-corrected (Benjamini and Hochberg 1995). All analyses were conducted in MATLAB.

## 3 Results

### 3.1 Demographic information

One patient was excluded due to hand tremor during the MRI session, resulting in uncorrectable motion artifacts, and one healthy control was excluded due an incomplete MRI session. Demographic analysis showed no significant differences in age (*P* = 0.16) or sex (*P* = 0.60). Demographic and clinical details of the final cohorts are provided in Table 1.

**Table 1:** Demographical and clinical characteristics of Parkinson’s disease patients and healthy controls.

	Parkinson’s disease patients (n = 29)	Healthy controls (n = 19)
Age (years)	63.76 (9.02)	59.68 (10.80)
Sex^a^ (number of females)	13	10
Disease duration^b^ (years)	3.69 (2.75)	
MDS-UPDRS II	7.72 (5.55)	
MDS-UPDRS III	31.03 (9.18)	
Hoehn and Yahr (n)		
Stage I	3	
Stage II	22	
Stage III	1	
Stage IV	0	

All values are presented as the mean, with standard deviation in parentheses unless otherwise specified.

a Sex was defined as the biological attribute assigned at birth (male/female) based on external anatomy. No participants identified as intersex or reported differences of sex development (DSD). Gender identity was not assessed in this study.

b Disease duration is considered from the time of official diagnosis.

### 3.2 Empirical non-reversibility/non-equilibrium

**Fig. 2:**
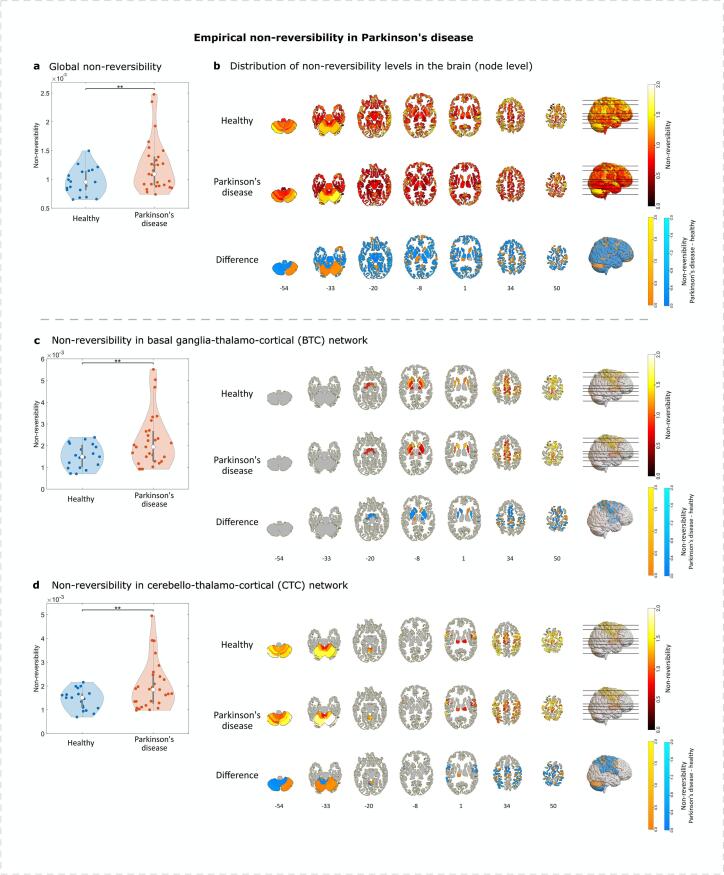
Empirical non-reversibility levels are higher in Parkinson’s disease (PD) across multiple spatial scales. (a) The global non-reversibility levels, computed using the model-free Arrow of Time technique across 1051 parcellated regions, are higher in PD compared to healthy subjects (*P* = 0.006), indicating a distortion in equilibrium. (b) On the node level, distribution of non-reversibility values across the brain in healthy and patient cohorts are visualized in numbered axial slices complemented by a 3D-rendered brain mapping on the right, with the difference (non-${reversibility}_{Parkinson^{\prime }s\;disease}-\;$non-${reversibility}_{Healthy})$ depicted below. Non-reversibility is elevated in PD, as revealed by the linear mixed model analysis (*P* = 0.010). (c-d) Left: increased non-reversibility values in the BTC and CTC networks in PD respectively (*P* = 0.007, *P* = 0.008); right: distribution of non-reversibility values and the difference between healthy and PD cohort across the brain in the BTC and CTC networks respectively. Visualization and 3D mapping were performed using MATLAB and MRIcroGL (Rorden and Brett 2000).

We analyzed empirical non-reversibility measures at global, network, and node levels, comparing PD to healthy states. Globally, the 1051 × 1051 non-reversibility matrices, averaged across nodes, exhibited higher levels in PD (*P* = 0.006; Fig. 2a). Results remained significant after outlier removal using the median absolute deviation (MAD) method (*P* = 0.031) (Leys et al. 2013). Network-level analysis of BTC and CTC, regions implicated in PD motor symptoms, also revealed significantly higher non-reversibility in PD (*P* = 0.007 and *P* = 0.008, respectively; Fig. 2c-d).

**Fig. 3:**
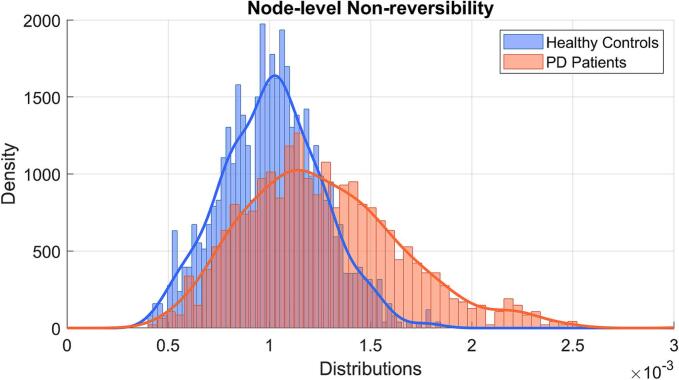
**Distribution of node-level non-reversibility values (averaged across subjects) in PD and healthy controls.** Histograms and overlaid kernel density estimates (KDE) show broader and right-shifted distributions in PD, consistent with increased variability observed in the Brown–Forsythe test of variance (P < 0.001). Each distribution reflects 1051 cortical and subcortical nodes per group.

Node-level analysis revealed significantly higher non-reversibility values in PD (estimate = 0.003, SE < 0.001, t = -2.553, *P* = 0.010). The distribution of node-level non-reversibility values was significantly broader in PD, as shown in Fig. 3, which presents overlaid histograms and kernel density estimates (KDE) based on values averaged across subjects. Formal distributional comparisons confirmed significant group differences: the MMD^2^ statistic indicated measurable divergence between groups (MMD^2^ = 0.00012), and the minimum energy test yielded a statistically significant result (*P* = 0.023). These findings suggest that PD is associated with a shift in the overall spatial distribution of non-reversibility across brain regions.

To benchmark our approach against more conventional methods, we repeated the main analysis using FC matrices across all scales. Significantly higher FC values were observed in PD at the node level (*P* < 0.001), but no differences were found on the global (*P* = 0.150) or network levels (BTC: *P* = 0.396; CTC: *P* = 0.148).

Testing the association between non-reversibility measures and clinical severity in PD patients yielded no correlations with MDS-UPDRS III (global: *P* = 0.891; CTC: *P* = 0.891; BTC: *P* = 0.891) or Hoehn and Yahr stage (global: *P* = 0.787; CTC: *P* = 0.891; BTC: *P* = 0.891) at any spatial scale.

### 3.3 Model-based framework of non-reversibility

#### 3.3.1 Generative effective connectivity patterns in PD and healthy state

Mode strengths within GEC were compared between the cohorts using LMM, revealing a non-significant trend toward lower connectivity in PD patients (estimate = 0.094, SE = 0.134, t = 0.701, *P* = 0.483). Age and sex showed non-significant effects (*P* = 0.810 and *P* = 0.183), while substantial individual variability was observed (random effects variance = 0.194, SD = 0.441). Results are visualized in Supplementary Fig. S1.

To test GEC informativeness, an SVM was used for pattern separation, achieving 100 % accuracy in distinguishing PD from healthy states. In contrast, alternative inputs such as SC and FC matrices produced lower accuracies of 52.3 % and 63.5 %, respectively. To evaluate the statistical significance of this result, we performed a dedicated permutation test using leave-one-out cross-validation (LOOCV) on PCA-reduced GEC features (20 components), with 1000 shuffled-label iterations. The observed LOOCV accuracy exceeded all accuracies obtained from the permutations, yielding a permutation-based *P* < 0.001, confirming that the result is unlikely due to chance. As a robustness check, we randomly interchanged 25 % of PD and HC labels, resulting in a 23.0 % drop in accuracy.

**Fig. 4:**
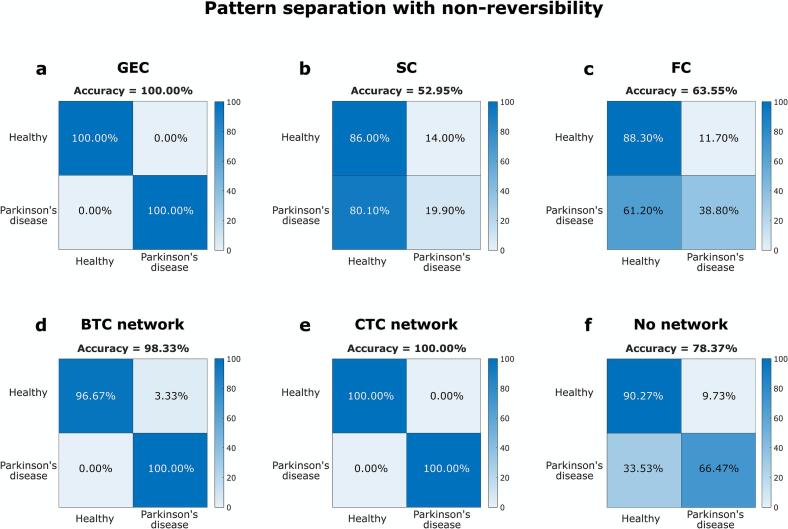
**Generative effective connectivity (GEC) patterns are distinct in Parkinson’s disease (PD)**. **(a)** SVM is able to separate whole-brain GEC patterns between PD and healthy states with 100% accuracy. **(b-c)** In contrast, pattern separation with alternative measures of structural connectivity (SC) and functional connectivity (FC) showed remarkably lower precision, emphasizing the informative quality of non-reversibility measures. **(d-e)** Pattern separation with network models of the BTC and CTC loops achieved high precision (98% and 100%, respectively). **(f)** Testing a third no-network model resulted in a precision drop to 78%, suggesting that while BTC and CTC networks are not exclusive drivers of the results, they are relatively more informative in PD. The SVM was employed for pattern separation rather than classification, given the limited sample size, which precludes generalizable classification outcomes.

At the network level, the SVM achieved high accuracies of 98.3 % and 100 % for the BTC and CTC network models, respectively. As a validity test, the SVM was applied to no-network models (regions outside BTC or CTC), achieving 78.4 % precision. Detailed SVM performance across all cases is shown in Fig. 4.

#### 3.3.2 Hierarchical organization in PD

In evaluating global coherence and hierarchical levels between PD and healthy states, our analyses encompassed global, regional, and network scales. Globally, coherence exhibited a non-significant decrease in PD (*P* = 0.25; Supplementary Fig. S2). Node-level hierarchical indices, analyzed via LMM, similarly showed no significant differences between cohorts (estimate = 0.002, SE = 0.003, t = 0.813, *P* = 0.415).

**Fig. 5:**
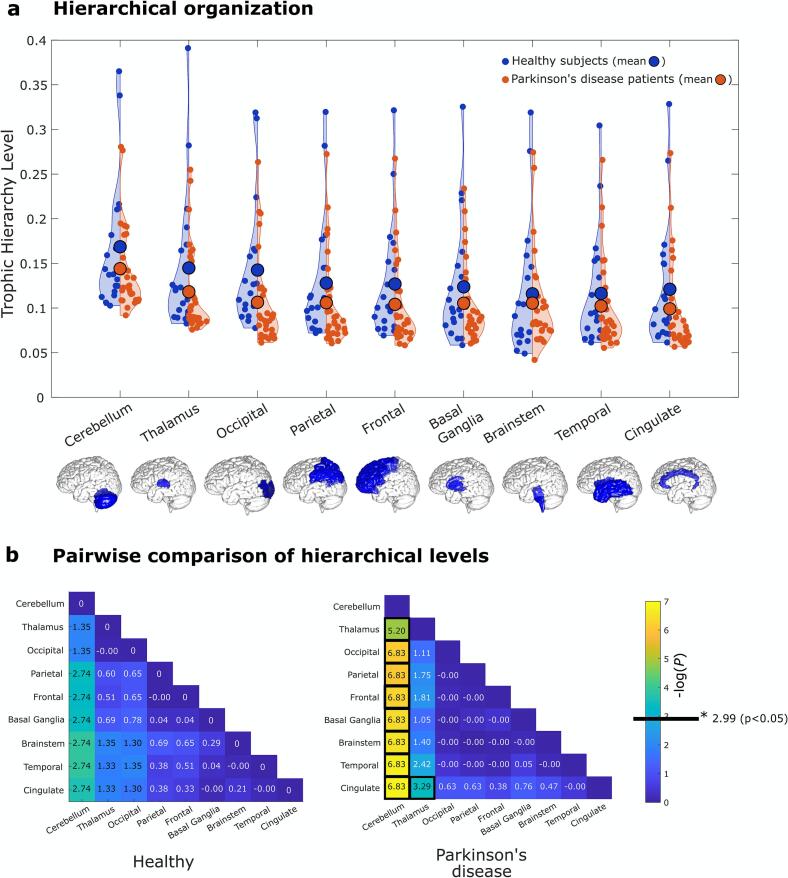
**Functional hierarchical organization in Parkinson’s disease (PD)**. Hierarchical levels are computed based on information in- and out-flow symmetry in the directed graph of whole-brain generative effective connectivity (GEC) models. **(a)** Hierarchical organization across major brain regions revealed lower hierarchy indices in PD, albeit not significant. PD is associated with a flatter hierarchnical organization, as shown by a reduction in standard deviation in hierarchical ranks (*P* = 0.033). **(b)** Pairwise comparison of hierarchy levels between large regions reveals relatively higher hierarchical positions for the cerebellum and thalamus in PD, with *P*-values represented as −log(*P*) to facilitate visualization. The bold black line delineates the significant threshold, reflected by *P*-values < 0.05, and accordingly, in the matrices, the significant values are outlined by black boxes.

At the regional level, hierarchical indices showed an overall drop in PD, although not significantly different from healthy controls (Fig. 5a). Variance analysis revealed that the hierarchical organization is flatter in PD (*P* = 0.033). Healthy controls had a mean variance of 6.8850 (STD = 0.0008), while PD patients showed a mean variance of 7.1118 (STD = 0.0006).

Pair-wise comparisons exposed significant differences in hierarchical relationships, with the cerebellum and the thalamus raking notably higher in PD. Additionally, the height difference between the thalamus and cingulate cortex became significant in the disease state. The exact p-values for these comparisons are provided in Fig. 5b. Of note, the cerebellum also exhibited a relatively high PageRank centrality measure in the whole-brain directed GEC graph (Supplementary Fig. S3).

## 4 Discussion

Our results showed that PD shifts brain dynamics toward higher levels of non-reversibility across global, node, and network scales. Generative computational models reflecting the quality of non-reversibility revealed distinct patterns in PD. Additionally, hierarchical organization was found to be altered in PD, displaying a flatter structure with significant changes in the relationship of the cerebellum and thalamus with other brain regions.

Significant deviations in non-reversibility and reduced equilibrium levels observed in PD suggest disruptions in the brain's information processing dynamics, even in the relatively early stages of PD (average disease duration in our study = 4 years). Due to the heterogeneity of manifestations and variability of methodologies, achieving consensus in the literature on PD brain dynamics has been challenging (Darbin et al. 2013). However, the observed equilibrium disruption supports the notion that PD impacts global brain dynamics and disrupts the functional and dynamical coordination essential for maintaining healthy, flexible brain dynamics (Caligiore et al., 2016, Kim et al., 2017, Liu et al., 2021). Studies have shown that PD disrupts normal spatiotemporal synchronization, particularly in motor-related networks, impairing both movement coding and execution (Sharott et al., 2018, West et al., 2018). Specifically, electroencephalography studies have identified increased non-reversibility in PD, indicating global disorder in information processing (Zanin et al. 2020). Additionally, employing graph theory and alternative resting-state fMRI methods have revealed increased chaos in the whole-brain information processing, which can serve as a potential marker for pathologically altered brain dynamics in PD (Ghasemi and Mahloojifar, 2013, Kim et al., 2017).

It is important to clarify that our study does not aim to estimate entropy directly. Instead, our primary objective is to quantify temporal non-reversibility—specifically, the asymmetry between forward and time-reversed lagged dependencies in BOLD signals. This measure captures violations of temporal symmetry in brain dynamics and has been shown to reflect meaningful alterations in brain state in prior work (Deco et al., 2022, Cruzat et al., 2023, de la Fuente et al., 2023).

We observed a broader and right-shifted distribution of node-level non-reversibility values in PD compared to healthy controls, indicating both an overall increase and greater heterogeneity across brain regions. This suggests that PD disrupts the balance of temporal dynamics unevenly across the brain, with some regions affected more strongly than others. Such spatially heterogeneous alterations in non-reversibility are conceptually linked to impaired large-scale functional integration and adaptive network coordination in PD even at the earlier stages (Kim et al., 2017, Zanin et al., 2020, Tinaz, 2021).

Importantly, we observed diminished temporal reversibility within the BTC and CTC loops—networks that are critically implicated in PD symptomatology (Caligiore et al., 2016, Duval et al., 2016, Dirkx and Bologna, 2022). This reduction, consistent with impaired sensorimotor integration in PD that leads to widespread disconnectedness across the brain, spans from altered dynamics at individual nodes to disruptions in their interactions and synchronization (Helmich et al., 2010, Göttlich et al., 2013).

These findings are in line with recent reports of altered brain dynamics in PD, including reduced metastability (Kim et al. 2017), impaired network switching (Liu et al. 2021), and decreased integration efficiency (Sorrentino et al. 2021), all of which point to a breakdown in large-scale coordination. Our observation of diminished reversibility offers a converging systems-level signature of these disruptions, grounded in non-equilibrium dynamics. While fundamentally distinct from molecular assays such as the α-synuclein seed amplification test (Shahnawaz et al., 2020, Iranzo et al., 2021), non-reversibility-based metrics may provide complementary information when incorporated into multimodal diagnostic pipelines.

By incorporating subject-specific SC, FC, and non-reversibility into GEC models and performing pattern separation, we demonstrated that reduced non-reversibility is a distinct feature of PD, supported by remarkable accuracy of SVM results. This distinction was evident in the superior performance of GEC models compared to traditional measures such as FC and SC. This emphasizes the robustness of arrow of time methods in capturing the distinctive dynamical characteristics of PD. It is crucial to note that our primary objective was not classification per se, given the limitation of our sample size, but rather to assess how well GEC patterns capture the distinctions between the PD and healthy states.

Our findings reveal a flatter hierarchical structure in PD, indicating a less defined functional differentiation and reduced global efficiency in information processing during rest. While earlier studies have linked a flattened hierarchy to symmetry and increased equilibrium (Deco et al., 2022, Deco et al., 2023a, Kringelbach et al., 2023), their measures have focused on top-down relationships. In contrast, the concept of functional hierarchy in our study focuses on interdependency and influence among regions, arising the brain’s self-organizational properties and aligning with Buzsáki's proposal of multiple dynamic structures operating in a coordinated organization to achieve rapid temporal solutions for efficient computation (Buzsáki 2009). Therefore, the observed flatness in our study does not imply symmetry but rather points to pathological over-synchronization or excessive feedback loops that create abnormal dependencies, which is well-documented in PD literature and leads to a range of motor and non-motor symptom (Wei et al., 2014, Kim et al., 2017, Liu et al., 2021, Sorrentino et al., 2021, Shettigar et al., 2022).

Our results revealed that the cerebellum occupied the highest functional hierarchy level in both healthy and PD cohorts, as evidenced by its elevated hub centrality within directed GEC graphs. This result reflects the cerebellum's important role in brain dynamics and highlights the necessity of integrating this region into whole-brain models. The cerebellum’s prominent influential rank implies a substantial dependency of other regions on its information-processing capabilities, a function supported by its extensive intrinsic functional topography and its widespread, reciprocal connections with the motor cortex, thalamus, and basal ganglia (Caligiore et al., 2017, Stoodley et al., 2021). Notably, the cerebellum’s influence appears to intensify in the PD state, as reflected in its elevated hierarchical rank and centrality. This finding resonates with the long-standing hypothesis that the cerebellum may play a compensatory role in supporting motor function in the face of basal ganglia dysfunction. In response to aberrant motor signals—whether characterized by hypokinesia or hyperkinetic tendencies—the cerebellum may become hyperactive in an effort to recalibrate and correct motor output (Lewis et al., 2013, Wu and Hallett, 2013, Mitoma et al., 2020, Sadeghihassanabadi et al., 2022, Li et al., 2023). While our results are compatible with this interpretation, they reflect cross-sectional observations of altered influence patterns at rest and do not imply any claims about disease progression. Although our inference of cerebellar compensation is grounded in GEC modeling and aligns with prior literature, it is based on indirect evidence. We did not include direct measures of dopaminergic integrity, such as DaT-SPECT or PET imaging, which would be valuable in validating the compensatory role of the cerebellum. We recommend that future multimodal studies integrate generative modeling with neurotransmitter-integrated imaging to clarify the neurochemical basis of cerebellar involvement in PD.

Additionally, the slight elevation in the thalamus’s hierarchical ranking beside the cerebellum in PD suggests widespread network disruptions within the BTC and CTC loops, supported by reports of over-synchronization between the two centered on the thalamus (Caligiore et al., 2016, Sadeghi et al., 2024), potentially stemming from impaired beta oscillations—a hallmark of dopamine depletion in PD. Elevated beta activity is linked to hierarchical dysfunction, destabilizing the brain’s functional organization and deviating information processing from its healthy state (Moran et al., 2011, Zittel et al., 2015, West et al., 2018, Cagnan et al., 2019, Reis et al., 2019). Collectively, they highlight widespread PD-related disturbances that impair both local and global coordination of brain activity.

A limitation of our study is the limited sample size, with a potential negative effect on the robustness of the SVM results. In addition, while we aimed to assess individual effective connectivity patterns, our models were initialized on averaged SC matrices across groups, which might limit the model’s specificity. To address these constraints, supplementary analyses were performed using alternative modalities including SC, FC, and no-network models. The cerebellum has been incorporated into various whole-brain brain network models in recent years (Palesi et al., 2020, Meier et al., 2022); however, our study advances this approach by integrating a fine-grained cerebellar parcellation alongside finely parcellated cortical and subcortical regions, enabling precise node-level analyses across the brain. While this allows for a detailed exploration of ROI roles, it introduces challenges with multiple comparisons, particularly when averaging hierarchy indices across regions of varying sizes. To address this, we used distinct spatial scales and *a priori* assumptions about pathological networks in PD.

In spite of the vast coverage of regions by our parcellation, certain regions whose functionalites are implicated by PD, such as substantia nigra (SN), were not included as they are not explicitly segmented in the Lausanne 2018 atlas. This reflects a general limitation of most standard fMRI-based parcellation schemes, as the SN is a small midbrain nucleus difficult to reliably resolve at conventional 3 T fMRI resolution. While we were unable to analyze the SN directly, our modeling framework captures its downstream functional consequences within the BTC and CTC networks, consistent with the hypothesized system-level effects of nigral degeneration. Future studies are encouraged to include dynamics of SN and other PD-relevant regions using ultra-high field imaging (e.g., 7 T) or quantitative susceptibility mapping (QSM) in the context of arrow of time analyses.

This study pioneers the exploration of deviations from brain equilibrium in PD through analysis of the temporal reversibility of neural dynamics. Future research is encouraged to employ larger cohorts and correlate specific symptom severities and task-based functional imaging with detailed mappings of dynamic distortions within PD motor networks. Such integrative approaches allow for enhanced classifications based on GEC models and may yield more precise diagnostic markers and inform targeted therapeutic strategies. Specifically, investigating how reversibility patterns evolve across temporal scales would be advantageous, particularly for uncovering whether asymmetries in information flow are scale-dependent and how equilibrium properties shift across slower intrinsic timescales. Such analyses could provide deeper insight into hierarchical time constants, temporal integration processes, and multiscale disruptions in PD. Furthermore, targeted *in silico* perturbations using individualized GEC models hold promise for informing novel brain stimulation techniques designed to restore balance by transitioning the brain state toward a healthier state (Saenger et al., 2017, Deco et al., 2018, Kringelbach and Deco, 2020).

## 5 Conclusions

To conclude, PD shifts the brain toward a state of diminished equilibrium across multiple spatial scales. Computational models grounded in measures of non-reversibility illuminate the intricate system-level pathophysiology of PD, revealing an associated flattening of the functional hierarchical organization. This flattening reflects a reduction in the efficiency of global information processing and compromises the coordination of movement. The important roles of the BTC and CTC networks in PD pathophysiology are underscored by their contributions to the distortion of whole-brain dynamics and equilibrium. Our study highlights the considerable potential of the arrow of time approach in PD research, enhancing our understanding of the disease by relying on measures of temporal reversibility. This innovative framework integrates structural, functional, and dynamic dimensions, offering robust tools for the identification of biomarkers across various stages of PD and advancing the potential for precise, holistic diagnostics.

## 6 Glossary

• *Arrow of time:* the directional progression of information processing within a system (such as the human brain), driven by the changes in entropy level and energy flow.
• *Equilibrium:* a state in which the system operates in perfect balance, with zero net flow of information or energy, leading to a stable condition where events occur symmetrically and without a definable direction in time. The healthy human brain functions at a certain level of non-equilibrium, characterized by energy-consuming and non-reversible neural processing and interactions.
• *Effective connectivity:* the directed dynamical interactions between brain regions, computable based on temporal reversibility, allowing for the analysis of brain functions and dysfunctions through generative models.
• *Functional hierarchical organization:* the functional arrangement of brain regions based on directional influence, where higher regions influence lower ones to maintain balance and energy efficiency. This hierarchy, characteristic of non-equilibrium systems, can be quantified by generative whole-brain models based on the arrow of time framework.
• *Hopf model (the arrow of time framework):* a generative computational framework that simulates brain dynamics by creating a whole-brain graph from individual structural connectivity. Each brain region is represented as a node equipped with Hopf oscillators, fitted and optimized using functional connectivity and temporal reversibility, making it effective for modeling complex neurological conditions such as Parkinson’s disease.
• *Parkinson’s disease:* a neurodegenerative disorder characterized by motor symptoms such as bradykinesia, postural instability, gait disorders, and tremor, resulting from the degeneration of dopamine-producing neurons in the substantia nigra. This leads to disrupted connectivity and desynchronization in networks such as the basal ganglia-thalamo-cortical (BTC) and cerebello-thalamo-cortical (CTC) networks.
• *Temporal reversibility:* Temporal reversibility is a quantifiable parameter that indicates the symmetry of information flow within the brain. In the healthy brain, temporal reversibility is detectable from the energy-dependent, non-reversible activities. Deviations from this measure can signal disruptions in normal brain function.

## CRediT authorship contribution statement

**Fatemeh Sadeghi:** Writing – review & editing, Writing – original draft, Visualization, Methodology, Investigation, Formal analysis, Data curation, Conceptualization. **Elvira del Agua Banyeres:** Writing – review & editing, Writing – original draft, Visualization, Methodology, Investigation, Formal analysis, Conceptualization. **Alessandra Pizzuti:** Writing – review & editing, Software, Resources, Methodology, Investigation, Formal analysis, Conceptualization. **Abdullah Okar:** Writing – review & editing, Data curation. **Kai Grimm:** Writing – review & editing, Data curation. **Christian Gerloff:** Writing – review & editing, Supervision, Resources, Project administration, Funding acquisition. **Morten L. Kringelbach:** Writing – review & editing, Validation, Supervision, Project administration, Methodology, Investigation, Funding acquisition, Formal analysis, Conceptualization. **Rainer Goebel:** Writing – review & editing, Visualization, Supervision, Software, Resources, Project administration, Funding acquisition. **Simone Zittel:** Writing – review & editing, Writing – original draft, Validation, Supervision, Resources, Project administration, Methodology, Investigation, Funding acquisition, Formal analysis, Conceptualization. **Gustavo Deco:** Writing – review & editing, Writing – original draft, Validation, Supervision, Resources, Project administration, Methodology, Investigation, Funding acquisition, Formal analysis, Conceptualization.

## Declaration of competing interest

The authors declare that they have no known competing financial interests or personal relationships that could have appeared to influence the work reported in this paper.

## Data Availability

Data will be made available on request.
