# Supplementary material for: The arrow of time in Parkinson’s disease
Source: Neuroimage Clin. 2025 Jun 24;47:103834. doi: 10.1016/j.nicl.2025.103834 (PMC12269992; doi:10.1016/j.nicl.2025.103834)
Supplement: Supplementary Data 1 [file mmc1.docx]

Supplementary Material

# Bridging parcellations

The MRI images were parcellated using the Lausanne 2018 atlas (Cammoun et al. 2012; Tourbier et al. 2022). In parallel, the cerebellum was segmented using the CERES pipeline (Romero et al. 2017; Carass et al. 2018) and added to the Lausanne parcellation, resulting in a combined parcellation denoted as LC. For the network-level analysis, the regions of interest listed in Table S1 were required to be marked and extracted from the LC parcellation. An issue however arises for the regions whose anatomical and functional delineations differ, and cannot be accurately extracted from the dominantly anatomical LC parcellation, including the Supplementary Motor Area, Primary Motor Cortex, Premotor Cortex, and Primary Somatosensory Cortex. These regions of interest are defined in the automated anatomical labeling3 (AAL3) (Rolls et al. 2020). parcellation regime and have unique Brodmann codes (Brodmann 1909). Therefore, the MRI images were in parallel parcellated with the AAL3/Brodmann atlas, then a custom-made MATLAB script (R2022b, The MathWorks, Inc.) was used to bridge the gap between the LC and AAL3/Brodmann parcellations. The algorithm loads parcellated and labelled volumes of one subject, then searches for specific AAL3/Brodmann labels in the LC parcellation and marks them. Having three sets of labels, in the analysis the most accurate label was chosen for each region of interest and used for masking and further network-based computations. Supplementary Table S1 includes details of regions, which network they belong to, and their corresponding AAL3 and Brodmann labels.

Supplementary Table 1. Regions of interest for network-level investigation of the arrow of time in Parkinson’s disease

| **Region of Interest** | **Network** | **LC Label** | **AAL3 Label** | **Brodmann Label** |
| --- | --- | --- | --- | --- |
| Basal Ganglia |  |  |  |  |
| Striatum | BTC | Caudate | Caudate nucleus | - |
|  |  | Putamen | Lenticular nucleus, Putamen |  |
| Globus pallidus externus (GPe) | BTC | Pallidum | Lenticular nucleus, Pallidum | - |
| Globus pallidus internus (GPi) | BTC |  |  | - |
| Subthalamic nucleus (STN) | BTC | Ventral Diencephalon(Neuromorphometrics 2005) | - | - |
| Thalamus |  |  |  |  |
| Ventral lateral anterior nucleus (VLa) | BTC | Ventro Latero Ventral | Ventral lateral | - |
| Ventral lateral posterior nucleus (VLp) | CTC | Ventro Latero Dorsal | Ventral posterolateral | - |
| Motor cortex |  |  |  |  |
| Supplementary Motor Area (SMA) | BTC | Superiorfrontal gyrus | Supplementary motor area | BA6 |
| Primary Motor Cortex (M1) | BTC | Precentral gyrus | Precentral gyrus | BA4 |
| Premotor Cortex (PMC) | CTC | Superiorfrontal gyrus | Superior frontal gyrus | BA6 |
| Primary Somatosensory Cortex (SMC) | CTC | Postcentral gyrus | Postcentral gyrus | BA1  BA2  BA3 |
| Cerebellum | CTC | 26 sub-regions (lobules, Crus, and Vermis) | 26 sub-regions (lobules, Crus, and Vermis) | - |

AAL = automated anatomical labelling; BTC = basal ganglia-thalamo-cortical network; CTC = cerebello-thalamo-cortical network

# The Hopf model

The Hopf model (Deco et al. 2017), as described below, represents the brain as a network of coupled oscillators. The local dynamics of each node are described by the normal form of a supercritical Hopf bifurcation. Each node $j$ is described by:

$\frac{dz_{j}}{dt}=z\left( a_{j}+i\omega_{j} -\left| z_{j}^{2} \right| \right)+g\sum_{k=1}^{N} C_{jk}\left( z_{k}-z_{j} \right)+\eta_{j}$ (S1)

where

$z_{j}=\rho_{j}e^{i\theta_{j}}=x_{j}+iy_{j}$ (S2)

and $\eta_{j}$ is additive Gaussian noise with standard deviation of $0.02$. The system has a bifurcation at $a_{j}=0$, so that for $a_{j}<0$ there is a stable fixed point at $z_{j}=0$ and for $a_{j}>0$ the dynamics exhibit limit cycle oscillations with a frequency of $\frac{\omega_{j}}{2\pi}$ Hz. We have used a fixed $a_{j}=-0.02$, and each node’s intrinsic frequency $\omega_{j}$is taken from the averaged peak frequency of the empirical time series of each brain region. Separating the real and imaginary parts of this equation yields:

$\frac{\partial x_{j}}{\partial t}=\left[ a_{j}-x_{j}^{2}-y_{j}^{2} \right]x_{j}-\omega_{j}y_{j}+\sum_{k=1}^{N} C_{jk}\left( x_{k}-x_{j} \right)+\eta_{j}$ (S3)

$\frac{\partial y_{j}}{\partial t}=\left[ a_{j}-x_{j}^{2}-y_{j}^{2} \right]y_{j}-\omega_{j}x_{j}+\sum_{k=1}^{N} C_{jk}\left( y_{k}-y_{j} \right)+\eta_{j}$ (S4)

The time series are modelled by the real variable $x_{j}$.

## Linearization of the model

Under the assumptions of small non-linearities and weak noise, the statistics of the whole system can be estimated using a linear approximation (Ponce-Alvarez and Deco 2024). Let bold letters denote column vectors and matrices. The dynamical system can be rewritten in vector form as

$\frac{d\boldsymbol{z}}{dt}=\left( \boldsymbol{a}-g\boldsymbol{S}+i\boldsymbol{\omega} \right) ⨀ \boldsymbol{z}-\left( \boldsymbol{z} ⨀ \bar{\boldsymbol{z}} \right)\boldsymbol{z}+g\boldsymbol{Cz}+\boldsymbol{\eta}$ (S5)

where $\boldsymbol{z}=\left[ z_{1}, \ldots,z_{N} \right]$, $\bar{\boldsymbol{z}}$ is the complex conjugate of $\boldsymbol{z}$, $\boldsymbol{a}=\left[ a_{1}, \ldots,a_{N} \right]$, $\boldsymbol{\omega}=\left[ \omega_{1}, \ldots,\omega_{N} \right]$, $\boldsymbol{S}=\left[ S_{1}, \ldots,S_{N} \right]$ contains the “strength” of each node $S_{i}=\sum_{j} C_{ij}$, and $\boldsymbol{\eta}=\left[ \eta_{1}, \ldots,\eta_{N} \right]$ represents a vector of uncorrelated noise. The symbol $⨀$ denotes the Hadamard product.

Linear fluctuations $\delta\boldsymbol{z}$ are studied here around the fixed point $\boldsymbol{z}=\boldsymbol{0}$, which is the solution of $\frac{d\boldsymbol{z}}{dt}=0$. We discard the higher-order terms $\left( \delta\boldsymbol{z}⨀\delta\bar{\boldsymbol{z}} \right)\delta\boldsymbol{z}$ and keep only the first-order terms of $\delta\boldsymbol{z}$. Let $\delta\boldsymbol{u}$ be a 2N-dimensional vector:

$\delta\boldsymbol{u}\mathbf{=(}\delta\boldsymbol{x}\mathbf{,}\delta\boldsymbol{y}\mathbf{)=}\left( {\delta x}_{1},\ldots,{\delta x}_{N},{\delta y}_{1},\ldots,{\delta y}_{N} \right)$ (S6)

Representing the evolution of the linear fluctuations. It follows the linear equation

$\frac{d}{dt}\delta\boldsymbol{u}=\boldsymbol{A}\delta\boldsymbol{u}\mathbf{+}\boldsymbol{\eta},$ (S7)

where the $2N\times2N$ matrix $\boldsymbol{A}$ is the Jacobian matrix of the system evaluated at the fixed point.

$A_{jk}=\left. \frac{\partial F_{j}}{\partial u_{k}} \right|_{\mathbf{0}},$ (S8)

where

$F_{j}=\left( a_{j}-{x_{j}}^{2}-{y_{j}}^{2} \right)x_{j}-\omega_{j}y_{j}+g\sum_{k=1}^{N} C_{jk}\left( x_{k}-x_{j} \right)$ (S9)

for $1\leq j\leq N$, and

$F_{j}=\left( a_{j}-{x_{j}}^{2}-{y_{j}}^{2} \right)y_{j}+\omega_{j}x_{j}+g\sum_{k=1}^{N} C_{jk}\left( y_{k}-y_{j} \right)$ (S10)

for $N+1\leq j\leq2N$.

The Jacobian matrix can be written as a block matrix by evaluating each of the partial derivatives:

$\boldsymbol{A}=\left[ \begin{matrix} \boldsymbol{A}_{\boldsymbol{xx}} & \boldsymbol{A}_{\boldsymbol{xy}} \\ \boldsymbol{A}_{\boldsymbol{yx}} & \boldsymbol{A}_{\boldsymbol{yy}} \end{matrix} \right]$ (S11)

Where $\boldsymbol{A}_{\boldsymbol{xx}}$, $\boldsymbol{A}_{\boldsymbol{xy}}$, $\boldsymbol{A}_{\boldsymbol{yx}}$, $\boldsymbol{A}_{\boldsymbol{yy}}$ are $N\times N$ matrices are given as: $\boldsymbol{A}_{\boldsymbol{xx}}\boldsymbol{=}\boldsymbol{A}_{\boldsymbol{yy}}\text{ = diag}\left( \boldsymbol{a}-g\boldsymbol{S} \right)+g\boldsymbol{C}$ and $\boldsymbol{A}_{\boldsymbol{xy}}\boldsymbol{=}\boldsymbol{-A}_{\boldsymbol{yx}}\text{ = diag}\left( \boldsymbol{\omega} \right)$, which is a diagonal matrix whose diagonal is the vector $\boldsymbol{\omega}$.

The statistics of the linear system can be determined from the Jacobian matrix, which depends on all the parameters of the model. Given an initial condition $\delta\boldsymbol{u}\left( 0 \right)$ at $t=0$, the general solution of a stochastic linear system such as equation (S6) is given by:

$\delta\boldsymbol{u}\left( t \right)=\boldsymbol{e}^{t\boldsymbol{A}}\delta\boldsymbol{u}\left( 0 \right)+\int_{0}^{t} \boldsymbol{e}^{\left( t-s \right)\boldsymbol{A}}d\boldsymbol{W}(s)$ (S12)

where $d\boldsymbol{W}$ is a 2N-dimensional Wiener process, and $\boldsymbol{e}^{t\boldsymbol{A}}$ is the exponential matrix defined as:

$\boldsymbol{e}^{t\boldsymbol{A}}=\sum_{k=0}^{\infty} \frac{1}{k!}\left( t\boldsymbol{A} \right)^{k}\mathbf{=}\boldsymbol{I}\mathbf{+}t\boldsymbol{A}\mathbf{+}\frac{1}{2!}\left( t\boldsymbol{A} \right)^{2}\mathbf{+}\frac{1}{3!}\left( t\boldsymbol{A} \right)^{3}+\cdots,$ (S13)

where $\boldsymbol{I}$ is the identity matrix. The right-hand side of equation (S12) is the sum of the deterministic behavior plus a stochastic integral representing the diffusion due to noise.

The linearization is only valid if the origin $\boldsymbol{z}=\boldsymbol{0}$ is a stable solution for the system. The stability of the origin can be determined by checking that all eigenvalues of $\boldsymbol{A}$ have a negative real part. Let $\lambda_{j}$ be the eigenvalues of $\boldsymbol{A}$. Then, the origin is asymptotically stable if $\text{Re}\left( \lambda_{\text{max}} \right)<0$, where $\lambda_{\text{max}}$ is the eigenvalue with the largest real part.

## Network statistics

In the following, we derive the network statistics of the linear system. The network mean activity (first-order statistic) is trivial since $\boldsymbol{z}=\boldsymbol{0}$. The first interesting statistic is the covariance of the fluctuations around the origin, i.e., $\boldsymbol{C}_{\boldsymbol{v}}=\left\langle\delta\boldsymbol{u}{\delta\boldsymbol{u}}^{\boldsymbol{T}} \right\rangle$, where the superscript $T$ denotes the transpose operator. For a stochastic linear system such as equation (S6), the motion equation of the covariance matrix $\boldsymbol{C}_{\boldsymbol{v}}$ is given as:

$\frac{d\boldsymbol{C}_{\boldsymbol{v}}}{dt}=\boldsymbol{A}\boldsymbol{C}_{\boldsymbol{v}}+\boldsymbol{C}_{\boldsymbol{v}}\boldsymbol{A}^{\boldsymbol{T}}+\boldsymbol{Q}_{\boldsymbol{n}}$, (S14)

where $\boldsymbol{Q}_{\boldsymbol{n}}=\left\langle\boldsymbol{\eta}\boldsymbol{\eta}^{\boldsymbol{T}} \right\rangle$ is the covariance matrix of the noise. For uncorrelated noise, $\boldsymbol{Q}_{\boldsymbol{n}}$ is diagonal, i.e., $\boldsymbol{Q}_{\boldsymbol{n}}\boldsymbol{=}\sigma^{2}\boldsymbol{I}$. The stationary covariance matrix can be obtained by solving $\frac{d\boldsymbol{C}_{\boldsymbol{v}}}{dt}=0$, which leads to the following algebraic equation:

$\boldsymbol{A}\boldsymbol{C}_{\boldsymbol{v}}+\boldsymbol{C}_{\boldsymbol{v}}\boldsymbol{A}^{\boldsymbol{T}}+\boldsymbol{Q}_{\boldsymbol{n}}=\mathbf{0},$ (S15)

Equation (S15) is an algebraic Lyapunov equation that has a unique solution provided that $\boldsymbol{A}$ is asymptotically stable. The Lyapunov equation can be solved using the eigen-decomposition of the Jacobian matrix. Let $\boldsymbol{A = VD}\boldsymbol{V}^{\boldsymbol{-1}}$, where $\boldsymbol{D}$ is a diagonal matrix containing the eigenvalues of $\boldsymbol{A}$, denoted $\lambda_{i}$, and the columns of matrix $\boldsymbol{V}$ are the eigenvectors of $\boldsymbol{A}$. Multiplying equation (S15) by $\boldsymbol{V}^{\boldsymbol{-1}}$ from the left and by the conjugate transpose of $\boldsymbol{V}^{\boldsymbol{-1}}$, noted $\boldsymbol{V}^{\boldsymbol{-\dagger}}$, from the right we get:

$\boldsymbol{C}_{\boldsymbol{v}}=\boldsymbol{VM}\boldsymbol{V}^{-\dagger},$ (S16)

where the matrix $\boldsymbol{M}$ is given as: $M_{ij}={-\tilde{Q}}_{ij}/(\lambda_{i}+\lambda_{i}^{*})$ and $\tilde{\boldsymbol{Q}}=\boldsymbol{V}^{\boldsymbol{-1}}\boldsymbol{Q}_{\boldsymbol{n}}\boldsymbol{V}^{\boldsymbol{-\dagger}}$. A fast, stable numerical solution of equation (S15) can be obtained using the MATLAB function *lyap.m* that uses the Bartels-Stewart method based on the Schur decomposition of the matrix $\boldsymbol{A}$ (Bartels and Stewart 1972).

Moreover, knowledge of the Jacobian matrix and the stationary covariance gives the stationary lagged covariances of the state variables, defined as $\boldsymbol{C}_{\boldsymbol{v}}(\tau)=\left\langle\delta\boldsymbol{u}{(t+\tau)\delta\boldsymbol{u}(t)}^{\boldsymbol{T}} \right\rangle$. Using the general solution of the system given by equation (S12), we get:

$\boldsymbol{C}_{\boldsymbol{v}}\left( \tau\right)=\boldsymbol{e}^{\tau\boldsymbol{A}}\left\langle\delta\boldsymbol{u}{(t)\delta\boldsymbol{u}(t)}^{\boldsymbol{T}} \right\rangle=\boldsymbol{e}^{\tau\boldsymbol{A}}\boldsymbol{C}_{\boldsymbol{v}}\left( 0 \right),$(S17)

Where $\boldsymbol{C}_{\boldsymbol{v}}\left( 0 \right)=\boldsymbol{C}_{\boldsymbol{v}}$ is the covariance matrix (i.e., zero-lag).

In summary, in the linear approximation, the stationary instantaneous and lagged covariance matrices of the model can be obtained through algebraic operations including the Jacobian matrix, also in the presence of time delays.

# Comparing GEC Node Strengths

To analyze the characteristics of the generative effective connectivity (GEC) models, we assessed and compared the node strength in the GEC graphs constructed for Parkinson's disease and healthy cohorts. Specifically, we measured the node strength, which reflects the total weight of connections i.e. the cumulative effective connectivity weights per node. This analysis offers insights into the connectivity levels of the ROIs in each cohort. We calculated the average node strengths for each group and visualized these results in Fig. S1.


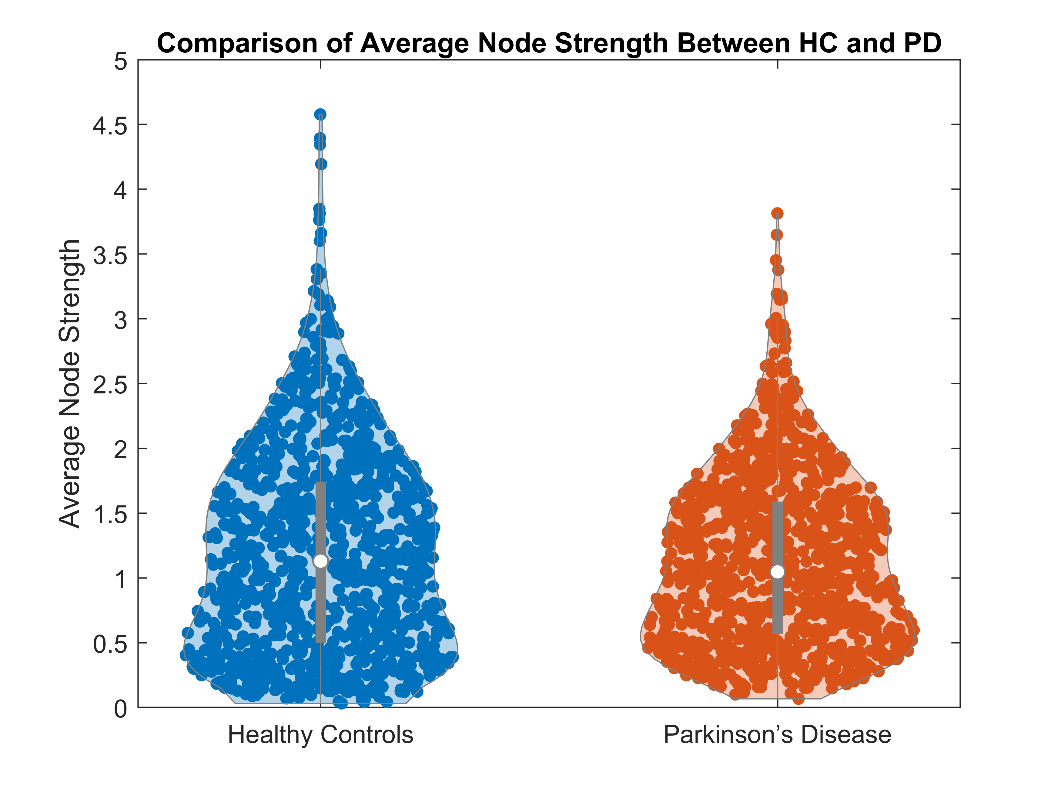


Figure S1. Comparison of average node strengths in GEC matrices between healthy and PD cohorts. A trend towards lower connectivity in PD compared to healthy control is visible, however further analysis with linear mixed models did not yield significant results.

To statistically analyze the differences in node strength between cohorts while controlling for individual variability, we constructed a linear mixed model (LMM). This model included node strength as the dependent variable, with cohort, age, and sex as fixed effect variables, and subject ID as a random effect. The analysis revealed that the effect of being in the healthy cohort relative to Parkinson’s disease was positive, indicating a trend towards lower connectivity in the disease state, however not statistically significant (estimate = 0.094, SE = 0.134, t = 0.701, P = 0.483). Age and sex effects were minimal and non-significant (P = 0.810 and P = 0.183, respectively). The random effects variance (0.194) and standard deviation (0.441) indicate substantial variability in node strength across individuals, accounted for in our model. These results suggest potential intrinsic network differences associated with disease status, albeit not significant.

# Global Coherence

Coherence level across the brain, borrowed from ecological models of trophic coherence, serves as a key measure for analyzing the hierarchical structure of the brain within whole-brain GEC models. This computational metric is derived from assessing the imbalance between in-flow and out-flow at each node, reflecting the functional coherence of the network. In practical terms, coherence signifies the degree of organization and efficiency within the brain's connectivity patterns, with maximally coherent networks exhibiting streamlined information flow. Conversely, higher levels of incoherence suggest greater disorganization or disruption in information processing, possibly indicating pathological conditions or alterations in brain function. In our investigation of coherence across Parkinson’s disease and healthy states, our findings revealed a non-significant decrease in overall coherence within the disease cohort compared to healthy controls (Fig. S2; *P* = 0.25).


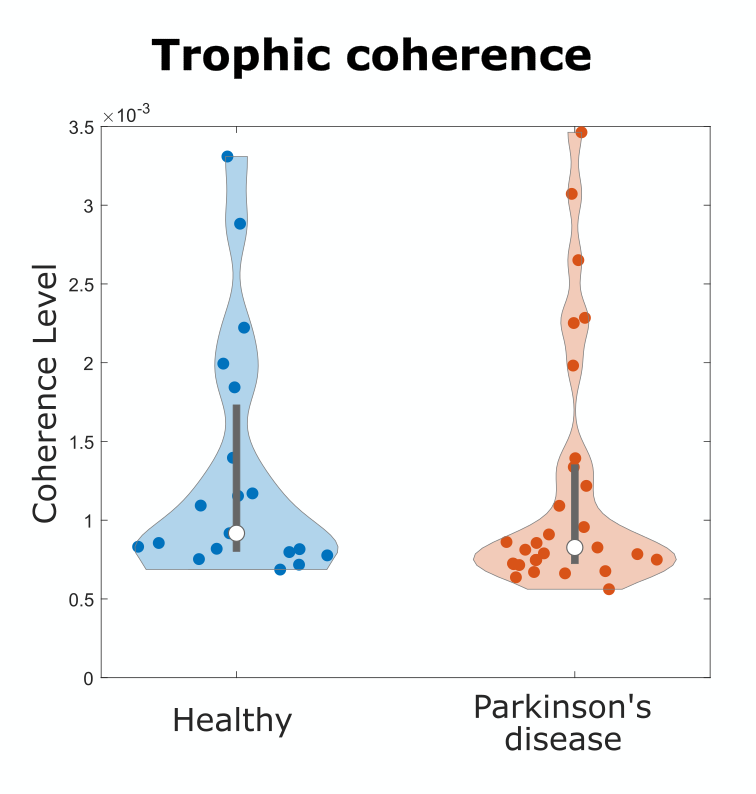


Figure S2. Global coherence in Parkinson’s disease and healthy state. Analyses reveal a decrease in overall coherence within the disease cohort compared to healthy controls, albeit not statistically significant (*P* = 0.25).

# Centrality measures in the Generative effective connectivity graph

To study the importance of each parcellated region in the grand scheme of generative effective connectivity (GEC) profiles, we followed the technique described by Mackay et al. (Mackay et al. 2020) and Ronen et al. (Ronen et al. 2014) to compute PageRank degrees of centrality for all nodes in the GEC directed graph and sort the nodes based on hierarchical ranks as well as centrality results. In Fig. S3 the whole brain GEC graph is plotted and the nodes with the highest degrees of PageRank centrality are marked, which also possess relatively high hierarchy levels. Certain cerebellar regions such as crus I, crus II, lobule VI, and lobule VIIIA possess relatively high levels in both the hierarchical organization PageRank centrality in both healthy and Parkinson’s disease states. Several sparsely located cortical gyri also exhibit higher centrality including superior frontal and parietal gyri in Parkinson’s disease and the same with the inclusion of precuneus and precentral gyri in the healthy states.


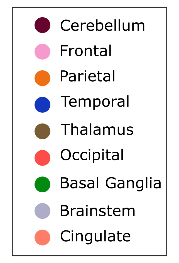

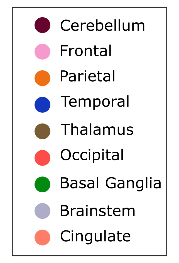

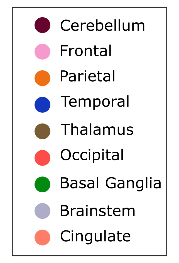

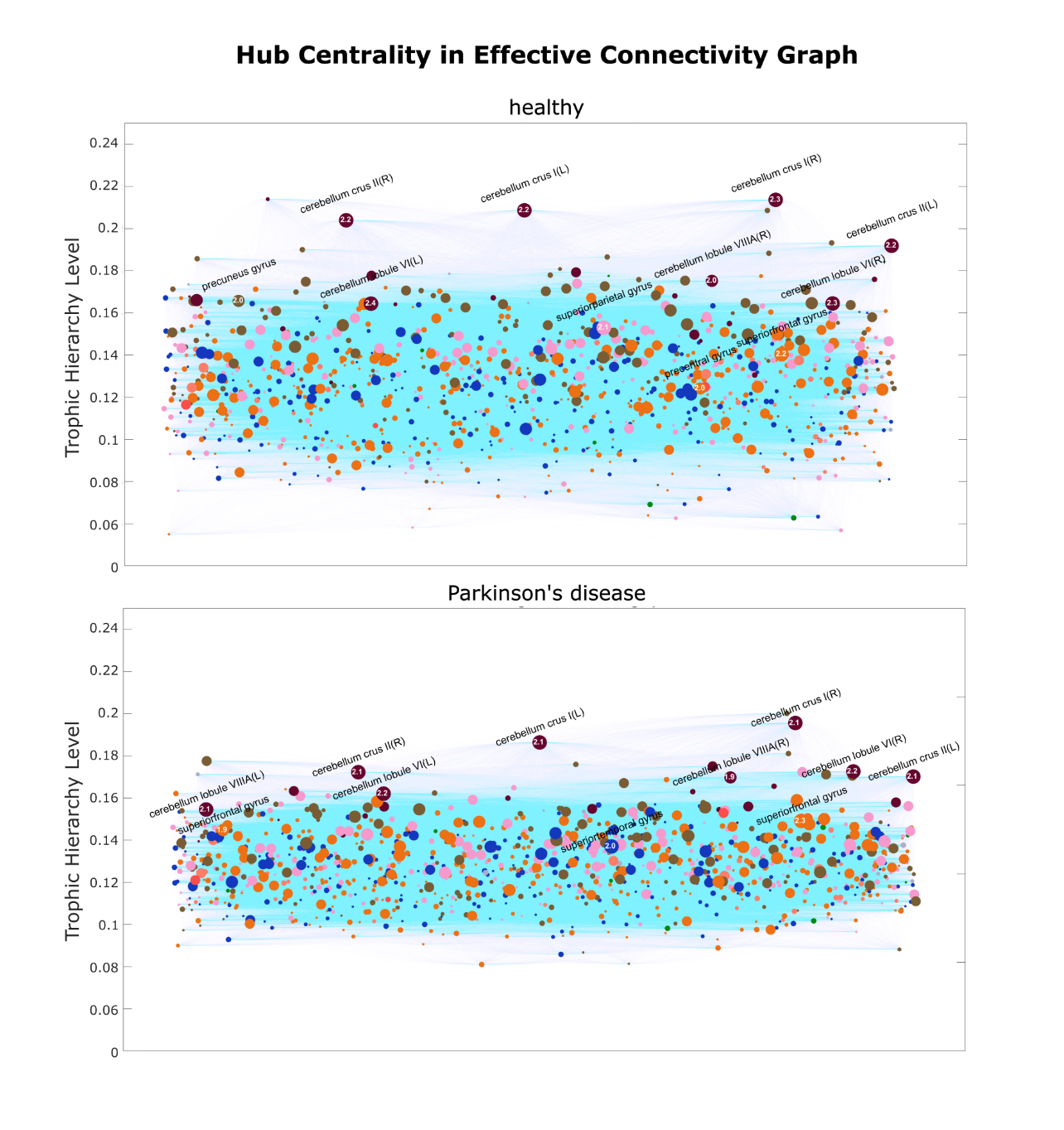


Figure S3. Hub Centrality Measures in Parkinson’s disease and healthy states. PageRank centrality measures were computed in the whole-brain directed graph of generative effective connectivity (GEC) profiles with 1051 nodes representing cortical, subcortical, and cerebellar regions; in both healthy and Parkinson’s disease states. The nodes with the top 1% with the highest centrality values were extracted and marked in the plot with their corresponding PageRank degree and region labels. Furthermore, hierarchical ranks were computed for each node based on the asymmetry of the in- and -outflow of that node (see Methods for further details) and viewed on the y-axis. The model-based approach of investigating non-reversibility combined with the principles of hierarchy analysis and graph theory can reveal how each region is categorized in terms of brain orchestration and flow of information. It can be seen from the plot that certain cerebellar regions such as crus I, crus II, lobule VI, and lobule VIIIA are relatively high in the hierarchical organization and possess higher degrees of centrality in both healthy and Parkinson’s disease states. A number of sparsely located cortical gyri also exhibit higher influence including superior frontal and parietal gyri in Parkinson, and the same with the inclusion of precuneus and precentral gyri in the healthy states. For the sake of visualization, the centrality measures were scaled and discretized into seven bins, and all node data points have been enlarged by a factor of 2 with the top 1% nodes further enlarged by a factor of 1.5.

# References

Bartels RH, Stewart GW. 1972. Algorithm 432 [C2]: Solution of the matrix equation AX + XB = C [F4]. Commun ACM. 15(9):820–826. doi:10.1145/361573.361582.

Brodmann K. 1909. Vergleichende Lokalisationslehre der Grosshirnrinde in ihren Prinzipien dargestellt auf Grund des Zellenbaues.

Cammoun L, Gigandet X, Meskaldji D, Thiran JP, Sporns O, Do KQ, Maeder P, Meuli R, Hagmann P. 2012. Mapping the human connectome at multiple scales with diffusion spectrum MRI. Journal of Neuroscience Methods. 203(2):386–397. doi:10.1016/j.jneumeth.2011.09.031.

Carass A, Cuzzocreo JL, Han S, Hernandez-Castillo CR, Rasser PE, Ganz M, Beliveau V, Dolz J, Ben Ayed I, Desrosiers C, et al. 2018. Comparing fully automated state-of-the-art cerebellum parcellation from magnetic resonance images. NeuroImage. 183:150–172. doi:10.1016/j.neuroimage.2018.08.003.

Deco G, Kringelbach ML, Jirsa VK, Ritter P. 2017. The dynamics of resting fluctuations in the brain: Metastability and its dynamical cortical core. Scientific Reports. 7(1):1–14. doi:10.1038/s41598-017-03073-5.

Mackay RS, Johnson S, Sansom B. 2020. How directed is a directed network?: How directed is a directed network? Royal Society Open Science. 7(9). doi:10.1098/rsos.201138.

Neuromorphometrics. 2005. Segmentation: Ventral Diencephalon. http://neuromorphometrics.com/Seg/html/segmentation/ventral diencephalon.html.

Ponce-Alvarez A, Deco G. 2024. The Hopf whole-brain model and its linear approximation. Sci Rep. 14(1):2615. doi:10.1038/s41598-024-53105-0.

Rolls ET, Huang CC, Lin CP, Feng J, Joliot M. 2020. Automated anatomical labelling atlas 3. NeuroImage. 206:116189. doi:10.1016/j.neuroimage.2019.116189.

Romero JE, Coupé P, Giraud R, Ta V-TT, Fonov V, Park MTM, Chakravarty MM, Voineskos AN, Manjón JV. 2017. CERES: A new cerebellum lobule segmentation method. NeuroImage. 147:916–924. doi:10.1016/j.neuroimage.2016.11.003.

Ronen S, Gonҫalves B, Hu KZ, Vespignani A, Pinker S, Hidalgo CA. 2014. Links that speak: The global language network and its association with global fame. Proceedings of the National Academy of Sciences of the United States of America. 111(52):E5616–E5622. doi:10.1073/pnas.1410931111.

Tourbier S, Rue-Queralt J, Glomb K, Aleman-Gomez Y, Mullier E, Griffa A, Schöttner M, Wirsich J, Tuncel MA, Jancovic J, et al. 2022. Connectome Mapper 3: A Flexible and Open-Source Pipeline Software for Multiscale Multimodal Human Connectome Mapping. Journal of Open Source Software. 7(74):4248. doi:10.21105/joss.04248.
